# Exposure to concentrated ambient particulate matter induces reversible increase of heart weight in spontaneously hypertensive rats

**DOI:** 10.1186/s12989-015-0092-6

**Published:** 2015-06-25

**Authors:** Zhekang Ying, Xiaoyun Xie, Yuntao Bai, Minjie Chen, Xiaoke Wang, Xuan Zhang, Masako Morishita, Qinghua Sun, Sanjay Rajagopalan

**Affiliations:** Department of Cardiology, East Hospital, Tongji University School of Medicine, Shanghai, 200120 People’s Republic of China; Department of Medicine Cardiology Division, University of Maryland School of Medicine, 20 Penn Street, Baltimore, MD 21201 USA; Division of Geriatric Medicine, Tongji Hospital, Tongji University School of Medicine, Shanghai, 200065 People’s Republic of China; Division of Environmental Health Sciences, College of Public Health, The Ohio State University, Columbus, OH USA; Department of Environmental Health Sciences, University of Michigan, Ann Arbor, MI USA

**Keywords:** PM_2.5_, Hypertension, Cardiac hypertrophy, Inflammation

## Abstract

**Background:**

Exposure to ambient PM_2.5_ increases cardiovascular mortality and morbidity. To delineate the underlying biological mechanism, we investigated the time dependence of cardiovascular response to chronic exposure to concentrated ambient PM_2.5_ (CAP).

**Methods:**

Spontaneously hypertensive rats (SHR) were exposed to CAP for 15 weeks, and blood pressure (BP), cardiac function and structure, and inflammations of lung, hypothalamus, and heart were measured at different time points.

**Results:**

Chronic exposure to CAP significantly increased BP, and withdrawal from CAP exposure restored BP. Consistent with its BP effect, chronic exposure to CAP significantly decreased cardiac stroke volume and output in SHR, accompanied by increased heart weight and increased cardiac expression of hypertrophic markers ACTA1 and MYH7. Withdrawal from CAP exposure restored cardiac function, weight, and expression of hypertrophic markers, supporting the notion that cardiac dysfunction and hypertrophy is subsequent to hypertension. In agreement with the role of systemic inflammation in mediating the cardiovascular effects of CAP exposure, chronic exposure to CAP markedly increased expression of pro-inflammatory cytokines in lung, heart, and hypothalamus. However, withdrawal from exposure resolves inflammation in the heart and hypothalamus, but not in the lung, suggesting that CAP exposure-induced systemic inflammation may be independent of pulmonary inflammation.

**Conclusion:**

Chronic exposure to CAP induces reversible cardiac dysfunction and hypertrophy, which is likely to be subsequent to the elevation in BP and induction of systemic inflammation as evidenced by increased mRNA expression of pro-inflammatory cytokines in diverse tissues.

## Background

An extensive body of scientific evidence indicates that long-term exposure to ambient PM_2.5_ is associated with premature death, especially related to cardiovascular diseases [1, 2]. The biological mechanisms underlying this association are however incompletely understood. One important gap in our knowledge regarding these biological mechanisms is the lack of information about the time dependence of cardiovascular response to ambient PM_2.5_ exposure, in particular repeated or chronic exposure that is common in the real world.

Hypertension, also known as high blood pressure, is a major risk factor for cardiovascular morbidity and mortality [3]. A recent meta-analysis has confirmed an association between exposure to ambient PM_2.5_ and increase in blood pressure (BP) [4]. Notably, in contrast to the large number of controlled human exposure and toxicological studies showing the elevation of BP in response to short-term exposure to ambient PM_2.5_, there are few studies investigating the BP effect of chronic exposure to ambient PM_2.5_. Most recently, we showed that chronic exposure to concentrated ambient PM_2.5_ (CAP) significantly increased BP in C57Bl/6j mice [5], likely through a central nervous system-dependent mechanism, supporting the hypothesis that the BP effect of chronic PM_2.5_ exposure may be associated with cardiovascular mortality. However, to verify the cause/effect relationship between them, a time dependency of BP response to chronic exposure to PM_2.5_ has yet to be determined.

Left ventricular hypertrophy (LVH), one of the most common complications of hypertension, has long been recognised as an important clinical prognostic entity and associated with increased cardiac morbidity and mortality [6]. So far, one epidemiological study already showed that living in close proximity to major roadways is associated with higher left ventricular mass index [7], suggesting that air pollution may also be associated with LVH in humans. We previously demonstrated that 12 weeks of exposure to CAP potentiated LVH of adult C57bl/6j mice in response to angiotensin II through RhoA/Rho kinase-dependent mechanisms [8], and one year of exposure to CAP alone was even sufficient to induce LVH in C57Bl/6j mice [9]. Consistent with this, Weldy et al. recently showed that exposure to diesel exhaust air pollution during *in utero* and early-life development in mice increases adult susceptibility to cardiac hypertrophy [10]. However, the same group also demonstrated that up to 6 months of diesel exhaust exposure had no effect on cardiac hypertrophy and heart function induced by angiotensin II stimulation or pressure overload in adult C57BL/6j mice [11], suggesting that further investigations on the association between ambient PM_2.5_ exposure and LVH are warranted.

In the present study, we exposed spontaneously hypertensive rats (SHR) to CAP, and investigated the time dependency of BP response to repeated exposure to CAP. To confirm the effect of CAP exposure on cardiac hypertrophy and test whether it is subsequent to hypertension, CAP exposure was withdrawn, and BP and cardiac function and hypertrophy were analysed at different time points.

## Results

### Exposure characterization

The average ambient daily PM_2.5_ concentration during the exposure period was 10.9 ± 3.6 μg/m^3^. The in-chamber average concentration during CAP exposure was 128.3 ± 60.4 μg/m^3^ versus 2.2 ± 1.3 μg/m^3^ for FA exposure. Since the exposures were performed for 6 h/day, 5 days/week, the 24-h average CAP concentration was 31.8 μg/m^3^, which was significantly higher than the annual national ambient air quality standard of 12 μg/m^3^ set by the U.S. Environmental Protection Agency (U.S. EPA 2012). Black carbon in ambient PM_2.5_ and CAP during the exposure period was 492.5 ± 283.4 and 5,269.5 ± 2,616.2 ng/m^3^, respectively. Table 1 shows the elemental composition of the ambient PM_2.5_ and CAP during the exposure period. Notably, if expressed as percentage of total mass, the elemental composition of CAP is almost identical to that of ambient PM_2.5_, confirming that our versatile aerosol concentration enrichment system did not significantly alter the chemical composition of ambient PM_2.5_. Correlation analysis of mass and trace element concentrations from 25 5-day samples of ambient PM_2.5_ (a superset which includes the 15-week inhalation exposure period) showed that the two highest correlations with PM_2.5_ mass concentrations were for sulfur (*R*^*2*^ = 0.91) and selenium (*R*^*2*^ = 0.84). Although identifying PM_2.5_ emission sources is generally beyond the scope of this paper, the strong, simultaneous correlations of sulfur and selenium suggest that the study site was most strongly affected by secondary aerosols, which are likely to include emissions from coal-fired utility boilers located regionally [12, 13].

**Table 1:** Elemental data of ambient PM_2.5_ and CAP

	Ambient PM_2.5_			CAP		
	Mean (ng/m^3^)	SD	%	Mean (ng/m^3^)	SD	%
Rb85	0.08	0.03	0.01	0.63	0.36	0.01
Sr88	0.39	0.11	0.04	3.34	1.63	0.04
Mo95	0.26	0.13	0.02	1.91	1.34	0.02
Cd111	0.27	0.21	0.03	2.27	2.18	0.03
Sb123	0.52	0.26	0.05	4.02	2.90	0.04
Ba137	2.63	1.32	0.25	22.60	16.19	0.25
La139	0.05	0.09	0.00	0.35	0.63	0.00
Ce140	0.04	0.01	0.00	0.32	0.17	0.00
Pb208	2.91	1.78	0.27	20.65	11.18	0.23
Na23	125.52	53.81	11.75	1081.31	600.46	12.08
Mg24	19.51	7.56	1.83	185.13	110.04	2.07
Al27	25.56	11.22	2.39	335.38	442.81	3.75
P31	7.63	4.32	0.71	132.38	158.12	1.48
S32	674.89	223.39	63.19	4760.37	3098.27	53.16
Ca44	95.73	35.93	8.96	1106.05	1092.73	12.35
Ti47	0.79	0.29	0.07	7.06	4.37	0.08
V51	0.15	0.07	0.01	1.08	0.73	0.01
Cr52	3.28	0.90	0.31	41.32	35.18	0.46
Mn55	1.56	0.71	0.15	13.63	8.21	0.15
Fe57	38.30	13.77	3.59	375.28	224.37	4.19
Co59	0.04	0.06	0.00	0.82	1.21	0.01
Ni60	2.67	9.58	0.25	256.58	1149.73	2.87
Cu63	2.84	1.15	0.27	39.17	51.35	0.44
Zn66	12.01	5.69	1.12	133.57	125.25	1.49
K39	49.53	16.76	4.64	422.51	219.53	4.72
As75	0.36	0.17	0.03	2.92	2.40	0.03
Se77	0.55	0.24	0.05	4.15	3.10	0.05

Ambient PM_2.5_ and CAP were collected weekly, and their elemental composition was determined as previously described [5]. The means and SDs of detectable elements and their percentages of ambient PM_2.5_ or CAP (%) are presented

### Exposure to CAP induces reversible increase in BP

**Fig. 1:**
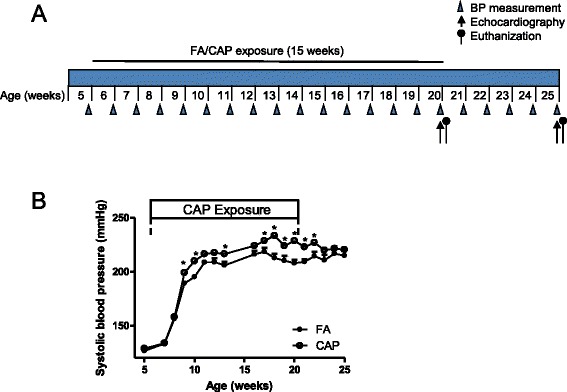
Exposure to CAP induces reversible increase in blood pressure. **a**, Experimental design: SHR were exposed to CAP for 15 weeks. Blood pressure was measured weekly. Following echocardiography in week 20 and week 25, rats were euthanized for myography and tissue harvesting. **b**, the blood pressure response of SHR to repeated CAP exposure and subsequent withdrawal. *p < 0.05 vs FA, two way ANOVA. *n* = 12 or 6/group

Figure 1 shows that 4 weeks of exposure to CAP was sufficient to significantly increased BP in SHR, and this significant difference in BP was observed throughout the remaining duration of exposure. To assess the time-dependency of this BP effects, after 15 weeks exposure, some rats were euthanized and the remaining were withdrawn from the exposure procedure. Figure 1 shows that the significantly increased BP in CAP-exposed animals was maintained only in 2 weeks after withdrawal from exposure to CAP.

### Exposure to CAP induces reversible vascular dysfunction

**Fig. 2:**
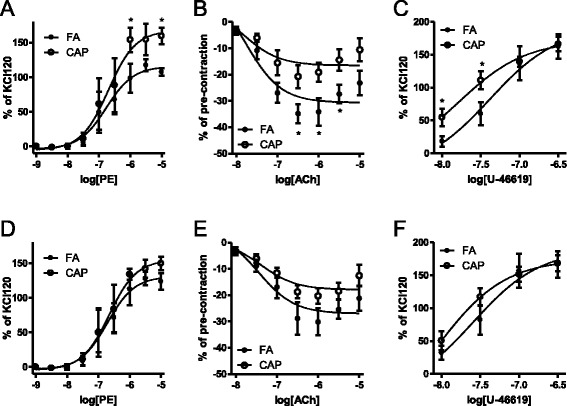
Exposure to CAP induces reversible vascular dysfunction. SHR were euthanized shortly after 15 weeks of exposure to CAP (**a**–**c**, week 20) or 5 weeks of withdrawal from CAP exposure (**d**–**f**, week 25). Thoracic aorta were harvested and mounted to myography as described in Methods. The responses of isolated thoracic aorta to the indicated concentration of phenylephrine (PE, **a** and **d**), acetylcholine (Ach, **b** and **d**), and U-46619 (**c** and **f**) were analyzed, and results are presented as mean ± SEM. *p < 0.05 vs FA, two way ANOVA. *n* = 6/group

A number of studies have demonstrated a potent effect of CAP exposure on vascular function [14], which may be implicated in the increased BP upon CAP exposure. Consistent with those studies, Fig. 2a–c show that exposure to CAP significantly increased the contractile responses of aortic rings to phenylephrine (a selective α1-adrenergic receptor agonist) and U-46619 (a stable synthetic analog of the endoperoxide prostaglandin PGH_2_), and significantly reduced the vasodilator action of acetylcholine. Furthermore, consistent with above results regarding BP, aortic contractile responses of SHR were almost completely restored after withdrawal from exposure to CAP for 5 weeks (Fig. 2d–f).

### Exposure to CAP induces reversible cardiac dysfunction and hypertrophy

**Fig. 3:**
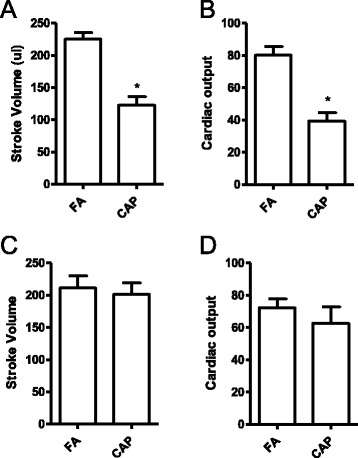
Exposure to CAP induces reversible cardiac dysfunction. SHR were subjected to echocardiography shortly after 15 weeks of exposure to CAP (**a** and **b**, week 20) or 5 weeks of withdrawal from CAP exposure (C and D, week 25). Stroke volume (**a** and **c**) and cardiac output (**b** and **d**) are presented. *p < 0.05 vs FA, student’s *t* test. *n* = 6/group

Adverse echocardiographic effects of exposure to CAP have previously been demonstrated in mice [9, 10]. To assess the effects of exposure to CAP on cardiac function of SHR, echocardiography was performed before euthanization of rats. Figure 3a and b show that 15 weeks of exposure to CAP significantly decreased stroke volume and cardiac output of SHR. No significant difference in other echocardiographic indexes was observed after 15 weeks of exposure to CAP (Week 20 in Table 2). After withdrawal from exposure to CAP for 5 weeks, the decreased stroke volume and cardiac output was completely restored (Fig. 3c and d and Table 2).

**Table 2:** Echocardiographic data

	Week 20		Week 25	
	FA	CAP	FA	CAP
HR (BPM)	355 ± 3.22	320.2 ± 8.9	367.4 ± 6.6	348.2 ± 8.8
Syst Diam (mm)	3.9 ± 0.17	4.3 ± 0.3	4.1 ± 0.3	4.2 ± 0.21
Dias Diam (mm)	7.4 ± 0.1	6.5 ± 0.2	7.3 ± 0.23	6.9 ± 0.25
Syst Vol (ul)	70 ± 7.45	98.5 ± 12.1	73.6 ± 14.11	85.4 ± 9.7
Dias Vol (ul)	295.3 ± 8.5	221.5 ± 1.35	291.3 ± 10.4	275.3 ± 11.2
Ejec Frac (%)	77.4 ± 1.67	61.2 ± 3.9	74.4 ± 3.51	69.4 ± 4.4
LVAW;d (mm)	2 ± 0.05	2 ± 0.07	2 ± 0.03	1.9 ± 0.07
LVAW;s (mm)	3.3 ± 0.07	2.7 ± 0.1	2.8 ± 0.09	3.1 ± 0.1
LVID;d (mm)	7.4 ± 0.1	6.9 ± 0.17	7.2 ± 0.09	6.9 ± 0.17
LVID;s (mm)	3.9 ± 0.13	4.2 ± 0.27	3.8 ± 0.2	4.2 ± 0.23
LVPW;d (mm)	2.5 ± 0.03	2.3 ± 0.1	2.3 ± 0.05	2.4 ± 0.09
LVPW;s (mm)	3.4 ± 0.02	2.9 ± 0.2	3.2 ± 0.1	3.1 ± 0.13
SV_Dias-Syst_ (ul)	226.4 ± 10.32	123.6 ± 11.9^*^	217.4 ± 15.21	190.2 ± 11.33
Frac Short (%)	48.4 ± 3.48	36.2 ± 2.9	46.2 ± 1.89	40.3 ± 2.47
LV Mass (mg)	1297 ± 246.8	1046 ± 229.4	1189 ± 219.7	1278 ± 347.7

Echocardiography was performed before euthanization and results are presented as mean ± SEM

*HR* heart rate, *Syst Diam* systolic diameter, *Dias Diam* diastolic diameter, *Syst Vol* systolic volume, *Dias Vol* diastolic volume, *Ejec Frac* ejection fraction, *LVAW;d* diastolic left ventricle anterior wall, *LVAW;s* systolic left ventricle anterior wall, *LVID;d* diastolic left ventricle internal dimension, *LVID;s* systolic left ventricle internal dimension, *LVPW;d* diastolic left ventricle posterior wall, *LVPW;s* systolic left ventricle posterior wall, *SV*_*Dias-Syst*_ stroke volume calculated by the difference between Dias Vol and Syst Vol, *Frac Short* fractional shortening, *LV mass* left ventricle mass

^*^ *p* < 0.05 versus FA, ANOVA

**Fig. 4:**
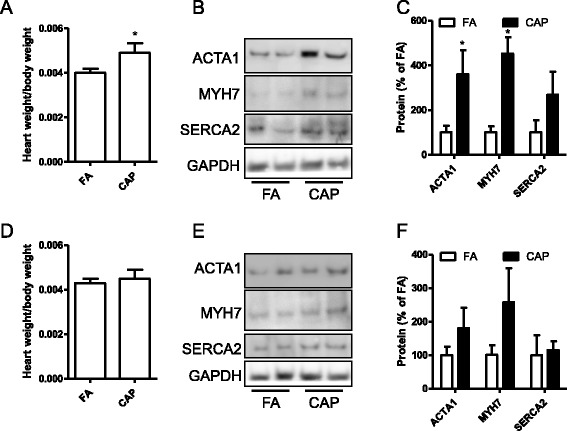
Exposure to CAP induces reversible cardiac dysfunction. **a**, the ratio of heart weight to body weight of SHR after 15 weeks of exposure to CAP (week 20). *p < 0.05 vs FA, student’s *t* test. *n* = 6/group. **b** and **c**, the cardiac protein levels of hypertrophic markers, ACTA1, MYH7, and SERCA2 after 15 weeks of exposure to CAP (week 20), as analysed by western blot. Representative images (**b**) and the summary (**c**) are presented. *p < 0.05 vs FA, one way ANOVA. D, the ratio of heart weight to body weight of SHR 5 weeks of withdrawal from CAP exposure (week 25). *p < 0.05 vs FA, student’s *t* test. *n* = 6/group. **d** and **e**, the cardiac protein levels of hypertrophic markers, ACTA1, MYH7, and SERCA2 5 weeks of withdrawal from CAP exposure (week 25), as analysed by western blot. Representative images (**d**) and the summary (**e**) are presented. *p < 0.05 vs FA, one way ANOVA

Cardiac hypertrophy is associated with BP and contributes to cardiac dysfunction and even mortality [6]. We previously showed that chronic exposure to CAP induced cardiac hypertrophy in C57Bl/6j mice [8]. To assess the effects of CAP exposure on cardiac mass of SHR, we weighed the body and organs of SHR. Figure 4a shows that 15 weeks of exposure to CAP significantly increased heart weight of SHR. In contrast, these CAP exposures did not significantly affect the weights of body and other organs (Table 3). ACTA1, MYH7, and SERCA2 are well-known markers for cardiac hypertrophy. Consistent with organ weight data, Fig. 4b and c show that exposure to CAP significantly increased the protein levels of ACTA1 and MYH7, reinforcing that exposure to CAP induced cardiac hypertrophy in SHR. Cardiac hypertrophy subsequent to hypertension has been shown to regress after treatment of hypertension [15]. Interestingly, Fig. 4e and f show that both heart weight and the expression of hypertrophic markers were restored after 5 weeks of withdrawal from exposure to CAP, supporting that exposure to CAP leads to cardiac hypertrophy probably through induction of hypertension.

**Table 3:** The weights of body and organs

Week	Body weight (g)		Liver weight (g)		Spleen weight (g)		Kidney weight (g)		Epi-Fat weight (g)	
	FA	CAP	FA	CAP	FA	CAP	FA	CAP	FA	CAP
20	322.1 ± 6.4	311.9 ± 14.9	14.7 ± 1.1	13.5 ± 1.1	0.64 ± 0.02	0.63 ± 0.12	1.5 ± 0.2	1.4 ± 0.2	1.3 ± 0.2	1.3 ± 0.2
25	348.4 ± 6.4	339.1 ± 17.6	15.4 ± 1.2	14.8 ± 1.4	ND	ND	ND	ND	2 ± 0.3	2.2 ± 0.6

The weights of body and the indicated tissues were measured during enthanization, and presented as mean ± SEM

### Exposure to CAP induces reversible inflammation

**Fig. 5:**
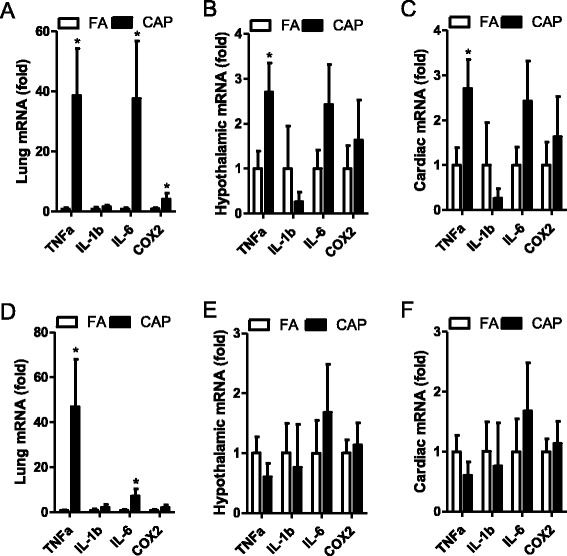
Exposure to CAP induces reversible local and systemic inflammations. SHR were euthanized shortly after 15 weeks of exposure to CAP (**a**–**c**, week 20) or 5 weeks of withdrawal from CAP exposure (**d**–**f**, week 25). The mRNA expression levels of indicated pro-inflammatory cytokines in lung (**a** and **d**), hypothalamus (**b** and **e**), and heart (**c** and **f**) were assessed by real-time RT-PCR. *p < 0.05 vs FA, one way ANOVA. *n* = 6/group

Systemic inflammatory response is believed to be subsequent to pulmonary inflammation and play a central role in the cardiovascular effects of chronic exposure to CAP [16]. Consistent with this notion, Fig. 5a–c show that 15 weeks of exposure to CAP significantly increased the mRNA expression of TNFα, IL-6, and COX2 in lung, and TNFα in heart and hypothalamus. Notably, while 5 weeks of withdrawal from CAP exposure restored the mRNA expression of TNFα in heart and hypothalamus (Fig. 5e and f), it did not restore the mRNA expression of TNFα and IL-6 in lung (Fig. 5d).

## Discussion

In the present study, we investigated the time dependency of cardiovascular responses to chronic exposure to CAP and subsequent withdrawal. The main findings include: 1) that the time course of BP response to CAP exposure reinforces a cumulative mode of action of repeated exposure to ambient PM2.5 on BP; 2) that chronic exposure to CAP induced cardiac dysfunction and hypertrophy in SHR, and both were restored by withdrawal from exposure to CAP. Along with the time course of BP response, our present data support that the cardiac effects of CAP exposure may be secondary to its BP effect; 3) that in tune with BP response, extra-pulmonary inflammation were induced by chronic exposure to CAP and were restored by withdrawal from exposure, replicating the notion that the BP effect of chronic CAP exposure may be mediated by systemic inflammation; 4) that chronic exposure to CAP induced both pulmonary and extra-pulmonary inflammation, and withdrawal from exposure restored extra-pulmonary but not pulmonary inflammation, suggesting that pulmonary inflammation may be central in initiation but not maintenance of extra-pulmonary inflammation.

Hypertension is a well-known major risk for cardiovascular morbidity and mortality [17]. A number of epidemiological and controlled exposure studies have indicated that exposure to ambient PM2.5 acutely increased BP in humans [18], suggesting that the acute BP response to PM2.5 exposure may contribute to its associated morbidity and mortality. Toxicological studies also show that short-term exposure to CAP increase BP in several animal models including dogs [19, 20], SHR [21], and WKY [22]. In the present study, we however did not observe an acute increase in BP in response to CAP exposure. This may be due to the difference in timing of BP measurement. While BP was measured during or immediately following exposure to CAP in those previous toxicological studies, it was measured 16 h after the last exposure to CAP in the present study. Interestingly, if taken together, these studies indeed suggest that the elevated BP induced by short-term exposure to PM2.5 may last for only a short duration, necessitating more time course study in the future on acute BP response to PM2.5 exposure.

In the present study, our data reveal that chronic exposure to CAP significantly increased BP in SHR. This is consistent with our previous study showing that chronic exposure to CAP increased BP in C57Bl/6j mice [5] and several epidemiological studies demonstrating associations between chronic exposure to PM2.5 and elevation in BP [4]. There is evidence that the effects of chronic CAP exposure on allergic response, atherosclerosis, and lung development follow a cumulative mode of action [23]. In contrast, the mode of action of chronic PM2.5 exposure on BP is not yet investigated. Interestingly, our data reveal a similar cumulative mode of action of chronic CAP exposure on BP, in particular in the term of the duration of elevation in BP: During the first 4 weeks of exposure, if there is any acute BP response, the duration of elevation in BP was less than 16 h; It was more than 16 h after 4 weeks of repeated exposure to CAP and was even up to 2 weeks after 15 weeks of exposure. To our knowledge, this is the first evidence that the BP effect of PM2.5 exposure may also follow a cumulative mode of action. Notably, the demonstration of this cumulative mode of action also somehow replicates that the aforementioned failure of identification of an acute BP response is indeed due to the timing of blood pressure measurement. Interestingly, consistent with several previous studies [24, 25], our data show that the BP effect of CAP exposure was paralleled by changes in vascular function (Fig. 2), suggesting that the CAP exposure-associated hypertension may be subsequent to vascular dysfunction.

LVH is a strong, independent predictor of cardiovascular events, including heart failure (HF) and all-cause mortality [26]. Epidemiological study has already demonstrated an association between chronic exposure to PM2.5 and LVH [7], and we previously showed that chronic exposure to CAP induced LVH in C57Bl/6j mice [8]. In the present study, we replicated that chronic exposure to CAP increased heart weight in SHR, which was accompanied by cardiac dysfunction, reinforcing that it may be pathologically important. LVH has diverse causes, including both environmental and genetic factors, and hypertension is one of the most common causes for LVH [26]. Since there is evidence that CAP exposure induces hypertension but absence of evidence that it changes other potential causes, it is likely that CAP exposure induces LVH through induction of hypertension. Hypertension-associated LVH has been shown to regress after success in control of BP [27]. Interestingly, our present data show that 5 weeks of withdrawal from CAP exposure was sufficient to restore CAP exposure-induced cardiac dysfunction and hypertrophy, reinforcing the notion that CAP exposure-induced LVH is secondary to hypertension. However, the exact role of hypertension in CAP exposure-induced LVH has yet to be determined through manipulation of blood pressure.

Systemic inflammation is generally believed to be the major mediator of the cardiovascular effects of chronic exposure to ambient PM2.5 [16]. Supporting this notion, our present data reveal that chronic exposure to CAP markedly increase pro-inflammatory cytokine expression in extra-pulmonary tissues including hypothalamus and heart, and the timing of increase in pro-inflammatory cytokine expression is in tune with those of hypertension and LVH: all were evident after 15 weeks of exposure to CAP, and were absent after 5 weeks of withdrawal from exposure. Notably, in spite of the importance of systemic inflammation in the toxicology of PM2.5 exposure, the mechanism whereby chronic exposure to CAP induces systemic inflammation remains unclear. In general, chronic PM2.5 exposure-induced extra-pulmonary inflammation is believed to be a spill-over of pulmonary inflammation, as previous time course studies demonstrated that pulmonary inflammation always precedes extra-pulmonary inflammation [16]. Interestingly, our present data show that 5 weeks of withdrawal from exposure to CAP was sufficient to resolve inflammation in hypothalamus and heart but not lung, clearly demonstrating that increased expression of some pro-inflammatory cytokines in lung subsequent to CAP exposure is not sufficient to maintain extra-pulmonary inflammation.

Our study, although providing important new findings, has some limitations. These include the fact that due to the limited space of exposure chamber, no comparison between rats withdrawn from exposure and those maintained on exposure was performed, which is important to confirm the effects of withdrawal from exposure to CAP. For the same reason, the present study did not include normotensive animals. Given that our previous study demonstrated that exposure to CAP increases BP in normotensive animals such as C57Bl/6j mice [5], it is warranted to determine if there is any difference between SHRs and their normotensive controls, WKYs, in the sensitivity to exposure to CAP. In addition, although together with previous demonstration of systemic inflammation-induced vascular dysfunction [28, 29], the restoring timings of PM2.5 exposure-induced systemic inflammation and vascular dysfunction in the present study support that PM2.5 exposure-induced vascular dysfunction results from systemic inflammation, the role of local vascular inflammation has yet to be determined.

## Conclusion

Chronic exposure to CAP induces reversible cardiac dysfunction and hypertrophy, subsequent to the elevation in BP and induction of systemic inflammation. In addition, our data suggest that CAP exposure induces systemic inflammation probably through pulmonary inflammation-independent mechanism.

## Methods

### Whole-body ambient inhalational CAP exposures protocol

The protocol of animal experiments was approved by the Ohio State University Institutional Animal Care and Use Committee, and all animals were treated humanely and with regard for alleviation of suffering. Animal exposure and the monitoring of exposure atmosphere and ambient aerosol were performed as previously described using a versatile aerosol concentration enrichment system that was modified for long-term exposures [8]. Briefly, SHR (4-week-old males, n = 6/group, 24 in total) were bought from Charles River and were housed in standard cages in a mobile trailer with a 12-hr light/12-hr dark cycle, temperatures of 65–75 °F, and relative humidity of 40–60 %. After 1 week of acclimation, rats were exposed to CAP or filtered air (FA) in chambers of the Ohio Air Pollution Exposure System for the Interrogation of Systemic Effects at The Ohio State University. All rats, including both FA and CAP groups, were exposed at exactly the same time. FA-exposed rats received an identical protocol with the addition of a high-efficiency particulate air filter (Pall Life Sciences, East Hills, NY, USA) positioned in the inlet valve to remove CAP in the filtered air stream, as previously described [8]. The exposure protocol comprised exposures for 6 h/day, 5 days/week (no exposure took place during weekends) for a total duration of 15 weeks.

### Sampling and analyses of ambient PM_2.5_ and CAP in the exposure chamber

To calculate exposure mass concentrations of ambient PM_2.5_ and CAP in the exposure chambers, samples were collected weekly on Teflon filters [Teflo, 37-mm, 2-μm pore (Pall Life Sciences, Ann Arbor, MI, USA)] and weighed before and after sampling in a temperature- and humidity-controlled weighing room using a Mettler Toledo no. 11106057 microbalance (Mettler Toledo, Columbus, OH, USA). The filters were then wetted with ethanol and extracted in 1 % nitric acid solution. The extraction solution was sonicated for 48 h in an ultrasonic bath and then allowed to passively acid digest for a minimum of 2 weeks. We then analyzed sample extracts for a suite of trace elements using inductively coupled plasma-mass spectrometry (ICP-MS) (ELEMENT2; Thermo Finnigan, San Jose, CA, USA) [30].

### Blood pressure assessment

The systolic blood pressure was obtained using a non-invasive tail-cuff monitor (MK2000; Muromachi Kikai, Tokyo, Japan). The measurement was performed weekly (Saturday morning). Before these analyses, all rats had been trained for 1 week. During assessment, the rats were placed into a holder which was pre-warmed and kept at 30 °C during the whole assessment and subjected to 20 measurements. The mean systolic blood pressure of these 20 measures for each animal were determined and presented.

### Echocardiography

Cardiac function was analysed by echocardiography (40 MHz transducer, Vevo 121 2100; Visualsonics, Toronto, Ontario), and the sonographer was blinded to the experimental groups during both data collection and analysis. Internal temperature was maintained at 37 °C as rats were continuously sedated with 1 % isoflurane (in 100 % O_2_) during assessment, which was about 1 h. Pre-warmed ultrasound gel (Aquasonic, Parker Labs, Fairfield, New Jersey) was used on the chest with a 15 MHz probe optimized for rats placed in the parasternal, short axis orientation. Data were averaged from at least three analyses per rat. LV dimensions (LVESd and LVEDd) and posterior wall thickness (PWTs and PWTd) were assessed, using the leading-edge technique according to the American Society for Echocardiography. Fractional shortening (FS) was calculated using the equation: % FS = [(LVEDd-LVESd)/LVEDd*100. Additional images were acquired to obtain aortic and pulmonary dimension, and pulse-wave Doppler imaging was used to obtain aortic and pulmonary velocities. Stroke volume (SV) was estimated utilizing the velocity time integral trace multiplied by the cross sectional area of the vessel, and this was used to calculate cardiac output (CO) as the product of SV and heart rate (HR). To determine the effects of withdrawal from CAP exposure on cardiac function, half rats were subjected to echocardiography shortly after CAP exposure, and the remaining were analysed after 5 weeks of withdrawal from CAP exposure.

### Tissue preparation

After anesthetized with pentobarbital sodium, rats were subjected to blood collection from hearts using a 22 g needle, and rapidly headed. Hypothalamus was isolated using incision sites as follows: rostral border of the optic chiasm, caudal border of the mamillary body, ventral border of the anterior commissure and lateral borders of the tuber cinereum and mamillary body complexes, and snap-frozen in liquid nitrogen. Thoracic aortas were proximally isolated and cut at the root. The whole heart was then isolated and placed on kimwipes to remove the remaining blood, and then snap-frozen in liquid nitrogen. All tissues were stored at −80 °C until processed.

### Myography

Briefly, rats were anesthetized with pentobarbital sodium, and the thoracic aorta was quickly removed and cleaned in physiological salt solution (PSS) containing (mM): NaCl, 130; NaHCO_3_, 14.9; KCl, 4.7; KH_2_PO_4_, 1.18; MgSO_4_•7H_2_O 1.18; CaCl_2_•2H_2_O, 1.56, EDTA, 0.026, glucose 5.5. The aorta was cut into 2-mm rings, and were then mounted in a muscle bath containing PSS at 37 °C and bubbled with 95 % O_2_-5 % CO_2_. Isometric force generation was recorded with a Multi Myography System (Danish Myo Technology A/S, Aarhus N, Denmark). A resting tension of 30 mN was imposed on each ring, and the rings were allowed to equilibrate for 2 h. Arterial integrity was assessed first by stimulation of vessels with 80 mM KCl. Endothelium-integrity was assessed by measuring the dilatory response to ACh (10 μM) in PE-contracted vessels (1 μM). To examine the contractility of those aortic rings, phenylephrine (PE) or U-46619 was added in a cumulative manner. To test relaxation responses, aortic rings were pre-contracted by PE (0.3 μM) that induced approximately 60 % of maximal contraction, followed by addition of acetylcholine (ACh) in a cumulative manner.

### Western blot analysis

About 1 g of left ventricle wall close to the apex of heart was cut on ice, and homogenized in M-PER Mammalian Protein Extraction Reagent (Pierce, Rockford, IL, USA). 40 μg/sample proteins were resolved with 10 % SDS-PAGE and transferred to membrane. Immuno-staining was performed by standard techniques with primary antibodies as follows: anti-GAPDH from Cell Signaling Technology (Boston, MA, USA); anti-ACTA1 from ABGENT (San Diego, CA, USA); anti-SERCA2 from Thermo Fisher Scientific (Rockford, IL, USA); and anti-MYH7 from GeneTex (Irvine, CA, USA). Signals were detected by supersignal™ chemiluminescence (Pierce, Rockford, IL, USA) and analysed by densitometry.

### Quantitative real-time reverse transcription polymerase chain reaction (RT-PCR)

Total RNA was isolated from tissues with TRIzol reagent (Invitrogen, Carlsbad, CA, USA). 4 μg total RNA was reverse transcribed using random hexamers and the ThermoScript RT-PCR System (Invitrogen). Quantitative RT-PCR was performed with the Stratagene Mx3005 using SYBER Green PCR Master Mix (Applied Biosystems, Carlsbad, CA, USA). The sequences of primers were previously described [31]: glyceraldehyde-3-phosphate dehydrogenase (GAPDH): sense, 5′-ATG ATT CTA CCC ACG GCA AG-3′, antisense, 5′-CTG GAA GAT GGT GAT GGG TT-3′; tumor necrosis factor-α (TNFα): sense, 5′-GAC CCT CAC ACT CA GAT CAT CTT CT-3′, antisense, 5′-TGC TAC GAC GTG GGC TAC G-3′; interleukin-6 (IL-6): sense, 5′-CGA GCC CAC CAG GAA CGA AAG TC-3′, antisense, 5′-CTG GCT GGA AGT CTC TTG CGG AG-3′); IL-1β: sense, 5′-CCC TGC AGC TGG AGA GTG TGG-3′, antisense, 5′-TGT GCT CTG CTT GAG AGG TGC T-3′; COX2: sense, 5′-GAT TGA CAG CCC ACC AAC TT-3′, antisense, 5′-CGG GAT GAA CTC TCT CCT CA-3′. The relative expression level was obtained as described previously with minor modification [8]. Briefly, Ct values were acquainted through analysis with software provided by the manufacturer, and differences of Ct value between target gene and GAPDH (∆Ct) and then 2^∆Ct^ were calculated. Results were expressed as ratio to the average of FA group (fold).

### Statistics

If not specified, data are expressed as mean ± SEM. Statistical comparisons of dose–response curves were performed with two-way repeated-measures analysis of variance (ANOVA) using GraphPad Prism version 4.0b (Graphpad Software Inc., La Jolla, CA, USA)]. Otherwise, statistical comparisons were performed with one-way ANOVA or Student’s *t*-test. p < 0.05 was considered to be statistically significant.

## Abbreviations

PM_2.5_: Particulate matter with aerodynamic diameter less than 2.5 μm
FA: Filtered air
BP: Blood pressure
CAP: Concentrated ambient PM_2.5_
LVH: Left ventricular hypertrophy
SHR: Spontaneous hypertensive rat
ACTA1: Actin, alpha 1
MYH7: Myosin, heavy chain 7, cardiac muscle, beta
SERCA2: Sarco/endoplasmic reticulum Ca^2+^-ATPase 2

## References

[1] Shah AS, Langrish JP, Nair H, McAllister DA, Hunter AL, Donaldson K (2013). Global association of air pollution and heart failure: a systematic review and meta-analysis. Lancet.

[2] Bell ML, Zanobetti A, Dominici F (2013). Evidence on vulnerability and susceptibility to health risks associated with short-term exposure to particulate matter: a systematic review and meta-analysis. Am J Epidemiol.

[3] van den Hoogen PC, Feskens EJ, Nagelkerke NJ, Menotti A, Nissinen A, Kromhout D (2000). The relation between blood pressure and mortality due to coronary heart disease among men in different parts of the world. Seven Countries Study Research Group. N Engl J Med.

[4] Liang R, Zhang B, Zhao X, Ruan Y, Lian H, Fan Z (2014). Effect of exposure to PM2.5 on blood pressure: a systematic review and meta-analysis. J Hypertens.

[5] Ying Z, Xu X, Bai Y, Zhong J, Chen M, Liang Y (2014). Long-term exposure to concentrated ambient PM2.5 increases mouse blood pressure through abnormal activation of the sympathetic nervous system: a role for hypothalamic inflammation. Environ Health Perspect.

[6] Frohlich ED, Gonzalez A, Diez J (2011). Hypertensive left ventricular hypertrophy risk: beyond adaptive cardiomyocytic hypertrophy. J Hypertens.

[7] Van Hee VC, Adar SD, Szpiro AA, Barr RG, Bluemke DA, Diez Roux AV (2009). Exposure to traffic and left ventricular mass and function: the Multi-Ethnic Study of Atherosclerosis. Am J Respir Crit Care Med.

[8] Ying Z, Yue P, Xu X, Zhong M, Sun Q, Mikolaj M (2009). Air pollution and cardiac remodeling: a role for RhoA/Rho-kinase. Am J Physiol Heart Circ Physiol.

[9] Wold LE, Ying Z, Hutchinson KR, Velten M, Gorr MW, Velten C (2012). Cardiovascular remodeling in response to long-term exposure to fine particulate matter air pollution. Circ Heart Fail.

[10] Weldy CS, Liu Y, Chang YC, Medvedev IO, Fox JR, Larson TV (2013). In utero and early life exposure to diesel exhaust air pollution increases adult susceptibility to heart failure in mice. Part Fibre Toxicol.

[11] Liu Y, Chien WM, Medvedev IO, Weldy CS, Luchtel DL, Rosenfeld ME (2013). Inhalation of diesel exhaust does not exacerbate cardiac hypertrophy or heart failure in two mouse models of cardiac hypertrophy. Part Fibre Toxicol.

[12] Reff A, Bhave PV, Simon H, Pace TG, Pouliot GA, Mobley JD (2009). Emissions inventory of PM2.5 trace elements across the United States. Environ Sci Technol.

[13] Morishita MKG, Kamal AS, Wagner JG, Harkema JR, Rohr AC (2011). Source identification of ambient PM2.5 for inhalation exposure studies in Steubenville, Ohio using highly time-resolved measurements. Atmos Environ.

[14] Terzano C, Di Stefano F, Conti V, Graziani E, Petroianni A (2010). Air pollution ultrafine particles: toxicity beyond the lung. Eur Rev Med Pharmacol Sci.

[15] Drazner MH (2011). The progression of hypertensive heart disease. Circulation.

[16] Brook RD, Rajagopalan S, Pope CA, Brook JR, Bhatnagar A, Diez-Roux AV (2010). Particulate matter air pollution and cardiovascular disease: an update to the scientific statement from the American Heart Association. Circulation.

[17] Bauer UE, Briss PA, Goodman RA, Bowman BA (2014). Prevention of chronic disease in the 21st century: elimination of the leading preventable causes of premature death and disability in the USA. Lancet.

[18] Pedersen M, Stayner L, Slama R, Sorensen M, Figueras F, Nieuwenhuijsen MJ (2014). Ambient air pollution and pregnancy-induced hypertensive disorders: a systematic review and meta-analysis. Hypertension.

[19] Bartoli CR, Wellenius GA, Diaz EA, Lawrence J, Coull BA, Akiyama I (2009). Mechanisms of inhaled fine particulate air pollution-induced arterial blood pressure changes. Environ Health Perspect.

[20] Chang CC, Hwang JS, Chan CC, Wang PY, Cheng TJ (2007). Effects of concentrated ambient particles on heart rate, blood pressure, and cardiac contractility in spontaneously hypertensive rats during a dust storm event. Inhal Toxicol.

[21] Chang CC, Hwang JS, Chan CC, Wang PY, Hu TH, Cheng TJ (2004). Effects of concentrated ambient particles on heart rate, blood pressure, and cardiac contractility in spontaneously hypertensive rats. Inhal Toxicol.

[22] Ito T, Suzuki T, Tamura K, Nezu T, Honda K, Kobayashi T (2008). Examination of mRNA expression in rat hearts and lungs for analysis of effects of exposure to concentrated ambient particles on cardiovascular function. Toxicology.

[23] Integrated Science Assessment for Particulate Matter. In: EPA, editor. 2009. http://cfpub.epa.gov/ncea/cfm/recordisplay.cfm?deid=216546. Accessed on 17 June 2015.

[24] Wauters A, Dreyfuss C, Pochet S, Hendrick P, Berkenboom G, van de Borne P (2013). Acute exposure to diesel exhaust impairs nitric oxide-mediated endothelial vasomotor function by increasing endothelial oxidative stress. Hypertension.

[25] Ying Z, Xu X, Chen M, Liu D, Zhong M, Chen LC (2013). A synergistic vascular effect of airborne particulate matter and nickel in a mouse model. Toxicol Sci.

[26] Levy D, Garrison RJ, Savage DD, Kannel WB, Castelli WP (1990). Prognostic implications of echocardiographically determined left ventricular mass in the Framingham Heart Study. N Engl J Med.

[27] Dahlof B, Hansson L (1992). Regression of left ventricular hypertrophy in previously untreated essential hypertension: different effects of enalapril and hydrochlorothiazide. J Hypertens.

[28] Dooley LM, Washington EA, Abdalmula A, Tudor EM, Kimpton WG, Bailey SR (2014). Endothelial dysfunction in an ovine model of collagen-induced arthritis. J Vasc Res.

[29] Javeshghani D, Barhoumi T, Idris-Khodja N, Paradis P, Schiffrin EL (2013). Reduced macrophage-dependent inflammation improves endothelin-1-induced vascular injury. Hypertension.

[30] Morishita M, Keeler G, Wagner J, Marsik F, Timm E, Dvonch J (2004). Pulmonary retention of particulate matter is associated with airway inflammation in allergic rats exposed to air pollution in urban Detroit. Inhal Toxicol.

[31] You Z, Luo C, Zhang W, Chen Y, He J, Zhao Q (2011). Pro- and anti-inflammatory cytokines expression in rat’s brain and spleen exposed to chronic mild stress: involvement in depression. Behav Brain Res.

